# Childhood Sexual Abuse and Compulsive Sexual Behavior Among Men Who Have Sex with Men Newly Diagnosed with HIV

**DOI:** 10.1007/s10461-024-04438-4

**Published:** 2024-07-11

**Authors:** Monique J. Brown, Medinat Omobola Osinubi, Daniel Amoatika, Mohammad Rifat Haider, Sally Kirklewski, Patrick Wilson, Nathan B. Hansen

**Affiliations:** 1 Department of Epidemiology and Biostatistics, Arnold School of Public Health, University of South Carolina, 915 Greene Street, Columbia, South Carolina USA; 2 Arnold School of Public Health, South Carolina SmartState Center for Healthcare Quality, University of South Carolina, Columbia, South Carolina USA; 3 Rural and Minority Health Research Center, Arnold School of Public Health, University of South Carolina, Columbia, South Carolina USA; 4 Office for the Study on Aging, Arnold School of Public Health, University of South Carolina, Columbia, South Carolina USA; 5 Department of Health Policy and Management, College of Public Health, University of Georgia, Athens, Georgia; 6 ENRICH Lab, Yale School of Public Health, New Haven, Connecticut, USA; 7 Department of Psychology, University of California Los Angeles, Los Angeles, CA USA; 8 Department of Health Promotion and Behavior, College of Public Health, University of Georgia, Athens, Georgia

## Abstract

Childhood sexual abuse (CSA) continues to be a public health challenge. The prevalence of experiencing CSA is higher among men who have sex with men (MSM) than the general population. CSA has been linked to compulsive sexual behavior (CSB) among varying populations but has not been examined among MSM who were newly diagnosed with HIV. Therefore, the aims of this study were to assess the direct association between CSA and CSB among newly diagnosed MSM living with HIV, and to identify the potential mediating roles of depressive symptoms and emotion regulation in the association between CSA and CSB. The study was a secondary data analysis using data obtained from 2012 to 2017 from two community HIV clinics in New York City (*n* = 202). CSA was operationalized with questions asking about sexual abuse during childhood/adolescence. CSB was measured using the 13-item Compulsive Sexual Behavior Inventory (CSBI). Depressive symptoms were measured using the 20-item Centers for Epidemiologic Studies Depression (CES-D) scale and emotion regulation was measured using a 36-item Difficulties in Emotion Regulation Scale (DERS). Path analysis was conducted to determine the mediating role of depressive symptoms and emotion regulation in the association between CSA and CSB. There was a statistically significant association between CSA and CSB (β = 0.160; *p* = 0.019). There were statistically significant indirect associations between CSA, depressive symptoms, emotion regulation, and CSB (depressive symptoms β = 0.0.071; *p* = 0.010; DERS: β = 0.080; *p* = 0.006). Depressive symptoms were also correlated with emotion regulation (*r* = 0.596; *p* < 0.001). The relationship between CSA and CSB was significantly mediated by depressive symptoms and emotion regulation. Trauma-informed interventions addressing depressive symptoms and difficulties in emotion regulation may help to reduce CSB among MSM living with HIV.

## Introduction

HIV rates appear to have stabilized in the United States, with an 8% decrease in new HIV cases between 2014 and 2019 [1]. Still, approximately 1.2 million people are living with HIV in the United States and over 30,000 people received an HIV diagnosis in 2020. Although only about 2% of the U.S. population identify as men who have sex with men (MSM), in 2020, 71% of new HIV cases occurred among MSM [1–3]. Further, underrepresented racial and ethnic minority groups are disproportionately represented among those with new HIV diagnoses, with 37% of new diagnoses occurring among Black or African American gay and bisexual men and 32% among Hispanic MSM of any race [2]. MSM ages 24–34 years have the highest number of new HIV diagnoses compared to other age groups [2]. Further, approximately 15% of MSM living with HIV do not know their status, particularly among younger minoritized populations [2].

While prior research has suggested many social factors that appear to impact MSM’s sexual health and overall well-being, including racism, discrimination, HIV-related stigma, homophobia, lower income and educational levels, and higher rates of incarceration or unemployment [2, 4], an often overlooked factor is the experience of childhood sexual abuse (CSA). Research has linked CSA with increased odds of acquiring HIV among MSM [5, 6]. Further, CSA has been found to be associated with HIV risk behaviors, such as unprotected sex, multiple sex partners, and substance use co-occurring with sex [5, 7, 8]. Prevalence rates of CSA among MSM are estimated to range between 10 − 50% [5, 9]. In two large studies of MSM in the U.S., reported rates of CSA experienced by men enrolled ranged from 27.3% (*N* = 15,622) [9] to 39.7% (*N* = 4,295) [6] compared to an estimated 5–10% of males in the general population [10]. CSA increases the risk of adverse mental health outcomes that last into adulthood [11]. This is significant considering MSM are already at risk for disproportionate rates of negative physical and mental health outcomes. When considering the link between CSA and sexual compulsivity these negative outcomes are likely to compound.

Sexual compulsivity is defined as “difficulties in controlling inappropriate or excessive sexual fantasies, urges/cravings or behaviors that generate subjective distress or impairment in one’s daily functioning” [12]. Individuals with sexual compulsivity report negative consequences that result such as an impact on mental health (94%), emotionally hurting a loved one (88%), decreased ability to experience healthy sex (78%), financial impact (53%), contracting a sexually transmitted infection (39%), job loss (17%), and legal issues (17%) [13]. Prevalence rates for sexual compulsivity are estimated to be between 3 and 6% of the adult population of the United States [14]. Men tend to have higher rates than women [15, 16] and studies have shown that MSM tend to have higher rates than the general population [14, 16]. Prevalence rates for MSM have been estimated to range between 19% and 30% [17].

CSA is associated with higher sexual compulsivity in both men and women, with a greater association for men [18]. Additionally, men who experienced sexual abuse as children have a higher rate of sexual compulsivity than men who did not experience CSA [18]. For those who experienced CSA, sexually compulsive behaviors can develop as a way to cope with negative emotions such as low self-esteem, shame or anxiety [14]. Researchers have also found a link between CSA and HIV for MSM. A meta-analysis found MSM who reported CSA were also more likely to live with HIV [9]. Furthermore, in a sample of MSM living with HIV, those who scored higher on the sexual compulsivity scale also reported significantly more sexual behaviors that could lead to HIV transmission [19]. Psychosocial risk factors experienced by MSM include sexual compulsivity, CSA, depressive symptoms, intimate partner violence and polysubstance use, which may leave them more vulnerable to acquiring HIV [17, 20].

Among MSM, research has found links between sexually compulsive behaviors and mood disorders such as depression and anxiety [14, 21] and dissociation [22]. Additionally, research has shown men who have sexually compulsive behaviors are more likely to have lower self-esteem [19, 23]. The negative impact of sexually compulsive behaviors is evident in the existing literature. However, few studies, to date, have explored the psychological processes related to the development of sexual compulsivity [22]. One such factor is CSA and its potential mediating pathways involving depressive symptoms and difficulties in emotion regulation.

MSM experience higher levels of depression compared to their heterosexual counterparts [24–28]. Within the MSM community, those who use substances, are people of color, and are living with HIV are at a greater risk for depression [24, 29–31]. CSA is associated with depression in the general population and MSM with a history of CSA are more likely to experience depression than MSM without a CSA history [32, 33]. Depression has been associated with sexual risk behavior such as sexual compulsivity and condomless sex with multiple partners [17, 34, 35].

Emotion regulation is the intrinsic and extrinsic process of managing emotions through behavioral and cognitive processes [36]. Individuals who have experienced discrimination such as homophobia have more difficulty regulating emotions than those who have not experienced homophobia [37]. Difficulties with emotion regulation in MSM can lead to behaviors such as disordered eating, increased number of sexual partners, increased alcohol and substance use, and depression [38–41]. Other factors, such as dissociation, anger, and living with HIV, are linked to more frequent sexual thoughts and urges [22]. Additionally, individuals who have experienced CSA tend to use maladaptive emotional responses more frequently than adaptive responses [42]. Men who have experienced CSA tend to use the maladaptive emotional responses of expressive suppression, cognitive avoidance, and rumination more than adaptive coping responses such as acceptance, positive reappraisal, and social support seeking [43].

Considering the variables described above and interrelationships among these variables, it is possible that CSA may lead to CSB through depressive symptoms and difficulties in emotions regulation. However, little research has examined these potential psychosocial pathways leading from CSA to CSB, particularly among newly HIV diagnosed MSM. Therefore, the aims of this study were to, using secondary data: (1) Determine the association between CSA and CSB among MSM newly diagnosed with HIV; and (2) Assess the mediating role of depressive symptoms and difficulties with emotion regulation between CSA and CSB.

## Methods

### Data Source and Study Population

Data were obtained from the baseline assessment of a larger intervention study for MSM who had been newly diagnosed with HIV (within the past year). Data for the intervention study were collected from November 2012 to March 2017. The intervention built upon and adapted the Positive Choices intervention [44], which was a two-session risk reduction intervention for MSM living with HIV. We adapted it to focus on engaging in care for men recently diagnosed with HIV. This included changing the “risk reduction plan” to a “health enhancement plan” focused on achieving health goals, which included discussion of engagement in care, adherence to medication, and disclosure decision-making. We also added three booster sessions at three weeks, three months, and six months post intervention to check in on and troubleshoot the health enhancement plan. Participants were recruited from two community HIV clinics in New York city. Eligibility criteria included: received an HIV diagnosis within the past year at one of the clinics or after referral to one of the two clinics after being diagnosed at another site; began care at one of the clinics or allow access to medical records if receiving care at another site; and be at least 18 years old. All participants gave written informed consent. Approximately 73% of men who presented for screening were eligible and enrolled in the study (202 out of 275). Screening and baseline assessments were administered using audio computer assisted self-interview (ACASI). These assessments took approximately 90 min. Participants received $40 for completing the assessments. The Columbia University and Yale University Institutional Review Boards approved the study.

### Measures

*Childhood Sexual Abuse*. CSA was operationalized as experiencing unwanted sexual behaviors as a child or adolescent vs. not experiencing CSA. Items were taken from an adapted version of the Traumatic Experience Checklist, which has been validated in patients with psychopathology [45]. Examples of questions include: As a child/adolescent, did you ever experience unwanted sexual touching, or were you made to touch someone else in a sexual way? As a child/adolescent, did you have unwanted oral sex (someone putting their mouth on a sexual part of your body, such as your penis or anus, or having you put your mouth on a sexual part of their body) whether you were offered gifts, coerced, threatened, or forced? As a child/adolescent, did you have unwanted sexual intercourse (someone putting, or trying to put, their penis or another object in your anus, or having you put your penis or another object in their vagina or anus) whether you were offered gifts, coerced, threatened, or forced?

*Compulsive Sexual Behavior.* The 13-item Compulsive Sexual Behavior Inventory (CSBI) was used to measure CSB, which has been validated among people reporting CSB [46]. Items were summed to give an overall sum score. Examples of items included “How often have you had trouble controlling your sexual urges?” and “How often have you made pledges or promises to change or alter your sexual behavior?” with answers on a 5-point Likert-type scale ranging from Never (1) to Very Frequently (5). Total scores range from 13 to 65 with higher scores indicating greater compulsive sexual behavior. The standardized Cronbach’s alpha for the CSBI was 0.94.

*Depressive Symptoms.* The 20-item Centers for Epidemiologic Studies Depression (CES-D) scale was used to measure depressive symptoms and has been validated in the general population [47]. Items were summed to give an overall score. Examples of items included: “I was bothered by things that usually don’t bother me” and “I felt everything I did was an effort” with answers on a 4-point Likert-type scale ranging from 0 “Rarely or none of the time (less than 1 day)”to 3 = “Most or all of the time (5 to 7 days)” reflecting depressive symptoms in the past seven days. Four items, which measure positive affect were reverse scored. Total scores range from 0 to 60, with higher scores indicating greater depressive symptomatology. The standardized Cronbach’s alpha for the CES-D was 0.92.

*Difficulties in Emotion Regulation.* The Difficulties in Emotion Regulation Scale (DERS) is a 36-item questionnaire that measures emotion regulation and dysregulation among adults [48]. The measure assesses how participants recognize, understand, accept, modulate, and respond to their emotions. Examples of items in the DERS included: “I experience my emotions as overwhelming and out of control” and “I am confused about how I feel.” Participants rated each item on a 5-point scale according to how often the statement applied to themselves, with responses of 1 = “Almost never (0–10%),” 2 = “Sometimes (11– 35%),” 3 = “About half the time (36–65%),” 4 = “Most of the time (66–90%),” and 5 = “Almost always (91–100%).” Total scores ranged from 36 to 180 with higher scores indicating greater emotion dysregulation. The standardized Cronbach’s alpha for the DERS was 0.94.

*Potential Confounders*. We controlled for sociodemographic characteristics including age (18–34, 35–49, 50+), sexual orientation (gay/homosexual, bisexual, heterosexual), race/ethnicity (Black, White, Hispanic/Latino, Multiracial, Asian/Pacific Islander/Other), education (less than high school, high school, some college, Bachelors/postgrad), employment (employed, unemployed), and income ($0-$10,000, $10,000-$20,000, $20,000-$30,000, over $30,000).

### Analytic Approach

Descriptive statistics were used to determine the distribution of sociodemographic characteristics, CSA, and CSB among MSM newly diagnosed with HIV. Median and interquartile range (IQR) values were reported for depressive symptoms and difficulties in emotion regulation. We conducted non-parametric tests (Wilcoxon) to compare median values for depressive symptoms and difficulties in emotion regulation. We conducted path analysis (crude and adjusted for sociodemographic characteristics) to determine the association between CSA (exposure), depressive symptoms and difficulties in emotion regulation (mediators) and CSB (outcome). We obtained direct and indirect standardized estimates between CSA, depressive symptoms, difficulties with emotion regulation and CSB. Statistical significance was considered at *p* < 0.05. All analyses were conducted in SAS (Cary, NC) and Mplus (Los Angeles, CA).

## Results

Table 1 shows the distribution of sociodemographic characteristics, childhood sexual abuse (CSA) and compulsive sexual behavior (CSB) among men who have sex with men newly diagnosed with HIV. The majority of men were between the ages of 18 and 34, identified as gay or homosexual; and were unemployed. The median (IQR) of the CES-D was 18 (9, 29) and of the DERS was 79 (61, 103). There was a statistically significant difference in CSA status by time since diagnosis where those who had a time since diagnosis greater than six months had a higher prevalence of CSA compared to those who were diagnosed within six months or less. There were no statistically significant differences in CSA status by other sociodemographic characteristics. In addition, the median values of CES-D and DERS were higher among those who had a CSA history compared to those who did not. There was a statistically significant difference in CSB by employment status where those who were unemployed had a higher mean (SD) of compulsive sexual behavior [14.8 (9.8)] compared to those who were employed [11.4 (10.1)]. CSB was significantly correlated with CES-D (*r* = 0.459, *p* < 0.001) and DERS (*r* = 0.492, *p* < 0.001). Approximately 45% (*n* = 90) of participants were on ART and 54% (*n* = 49) of those who were on ART were 100% adherent.

**Table 1:** Distribution of Sociodemographic Characteristics, childhood sexual abuse and compulsive sexual behavior among men who have sex with men newly diagnosed with HIV

Characteristics	Overall Sample *N* (%)	CSA *N* (%)	No CSA *N* (%)	*P*-value	Compulsive Sexual Behavior Mean (SD)	*P*-value
**Age (Mean, SD)** 18–34 35–49 50+	32.0 (9.1) 141 (69.8) 49 (24.3) 12 (5.9)	76 (66.1) 31 (27.0) 8 (7.0)	65 (74.7) 18 (20.7) 4 (4.6)	0.408	17.9 (13.2) 16.9 (11.5) 14.3 (12.2)	0.592
**Sexual Orientation** Gay/Homosexual Bisexual Heterosexual	173 (86.5) 25 (12.5) 2 (1.0)	76 (88.4) 10 (11.6) 0 (0.0)	97 (85.1) 15 (13.2) 2 (1.8)	0.436	17.3 (12.2) 18.5 (15.7) 9.5 (9.2)	0.595
**Race/Ethnicity** Black White Hispanic/Latino Multiracial Asian/PI/Other	84 (41.8) 33 (16.4) 51 (25.3) 21 (10.5) 12 (6.0)	37 (43.0) 12 (14.0) 23 (26.7) 10 (11.6) 4 (4.7)	47 (40.9) 21 (18.3) 28 (24.4) 11 (9.6) 8 (7.0)	0.851	16.9 (12.7) 16.3 (14.1) 18.3 (12.7) 19.2 (10.5) 18.7 (13.5)	0.881
**Education** < High School High School Some College Bachelors/Postgrad	12 (6.0) 33 (16.3) 64 (31.7) 93 (46.0)	8 (9.2) 13 (14.9) 33 (37.9) 33 (37.9)	4 (3.5) 20 (17.4) 31 (27.0) 60 (52.2)	0.073	23.2 (10.6) 15.3 (14.5) 18.0 (11.7) 17.1 (12.9)	0.253
**Employment** Employed Unemployed	82 (40.6) 120 (59.4)	29 (33.3) 58 (66.7)	53 (46.1) 62 (53.9)	0.068	14.9 (12.8) 19.2 (12.4)	**0.020**
**Income** $0-$10,000 $10,000-$20,000 $20,000-$30,000 Over $30,000	98 (49.0) 35 (17.5) 21 (10.5) 46 (23.0)	47 (55.3) 13 (15.3) 8 (9.4) 17 (20.0)	51 (44.4) 22 (19.1) 13 (11.3) 29 (25.2)	0.503	18.1 (13.5) 16.7 (9.4) 22.4 (14.9) 14.5 (11.8)	0.158
**Time since Diagnosis** ≤ 6 months > 6 months	160 (79.2) 42 (20.8)	62 (71.3) 25 (28.7)	98 (85.2) 17 (14.8)	**0.016**	17.6 (12.5) 16.8 (13.6)	0.726
Depressive symptoms (Median, IQR)	18 (9, 29)	23 (13, 35)	15 (7, 23)	**< 0.001**	0.470^a^	**< 0.001**
DER (Median, IQR)	79 (61, 103)	89 (71, 112)	71 (56, 95)	**< 0.001**	0.500^a^	**< 0.001**

**Abbreviations** ART – Antiretroviral therapy; CSA – Childhood Sexual Abuse; DER – Difficulties in Emotion Regulation; SD – Standard Deviation

^a^Correlation coefficient

**Bolded** p-values are statistically significant at *p* < 0.05

There was a statistically significant association between CSA and CSB (β = 0.160; *p* = 0.019). However, this association attenuated to be not statistically significant after putting the mediators (CES-D and DERS) in the model. Table 2 shows the direct standardized estimates between CSA, CES-D, DERS and CSB among the study population. After adjusting for sociodemographic characteristics, CSA was positively associated with CES-D (β = 0.282; *p* < 0.001) and DERS (β = 0.234; *p* = 0.001). The mediators were also positively associated with CSB (CES-D: β = 0.252; *p* = 0.001; and DERS: β = 0.341; *p* < 0.001). Finally, depressive symptoms (CES-D) were correlated with difficulties in emotion regulation (DERS) (*r* = 0.596; *p* < 0.001).

**Table 2:** Direct standardized estimates between childhood sexual abuse, depressive symptoms, difficulties with emotion regulation and compulsive sexual behavior among men who have sex with men living with HIV

	Crude β	*p*-value	Adjusted β*	*p*-value
CSA → CSB	0.000	0.994	0.019	0.759
CSA → Depressive Symptoms	**0.295**	**< 0.001**	**0.282**	**< 0.001**
CSA → DER	**0.258**	**< 0.001**	**0.234**	**0.001**
Depressive → CSB Symptoms	**0.257**	**0.001**	**0.259**	**0.001**
DER → CSB	**0.339**	**< 0.001**	**0.347**	**< 0.001**
Depressive Symptoms and DER (r)	**0.601**	**< 0.001**	**0.596**	**< 0.001**

**Abbreviations** CSA – Childhood Sexual Abuse; CSB – Compulsive Sexual Behavior; DER – Difficulties in Emotion Regulation

*Adjusted effect estimates controlled for age (continuous), sexual orientation, race, education, employment, and income

**Bolded** estimates and p-values are statistically significant at *p* < 0.05

**Fig. 1:**
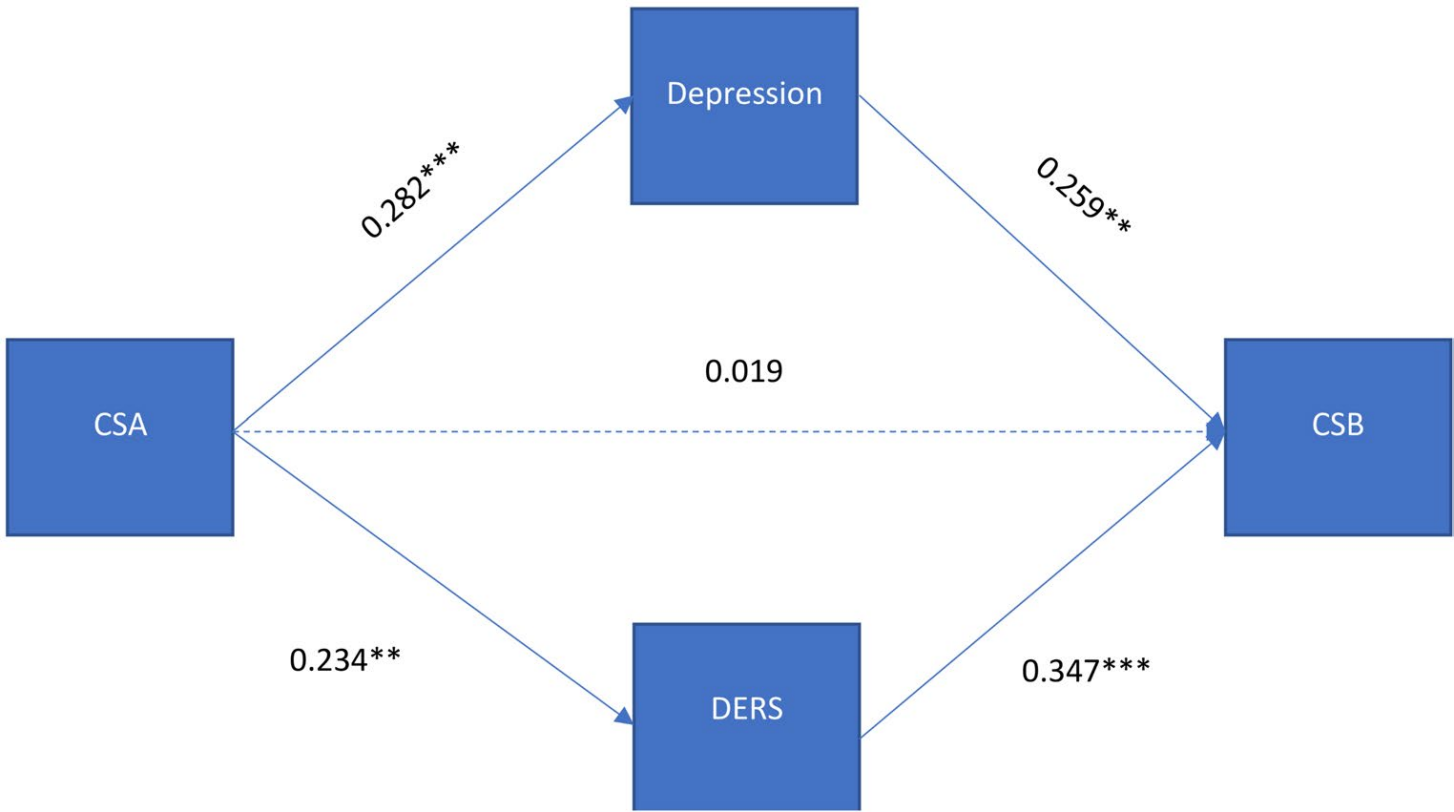
Mediating Pathway between Childhood Sexual Abuse, Depressive Symptoms, Difficulties in Emotion Regulation, and Compulsive Sexual Behavior in Demographically Adjusted Model. Note: ^***^p < 0.001, ^**^p < 0.01

Table 3 shows the indirect standardized estimates between CSA, CES-D, DERS and CSB among the study population. The findings suggest that depressive symptoms (CES-D; β = 0.071; *p* = 0.010) and difficulties in emotion regulation (DERS; β = 0.080; *p* = 0.006) fully mediated the association between CSA and CSB (Fig. 1). Crude and adjusted models had excellent fit. See Supplemental Table 1 for model fit indices.

**Table 3:** Indirect standardized estimates between childhood sexual abuse, depressive symptoms, difficulties with emotion regulation and compulsive sexual behavior among men who have sex with men living with HIV

	Crude β	*p*-value	Adjusted β*	*p*-value
Depressive Symptoms	**0.076**	**0.007**	**0.073**	**0.008**
DER	**0.088**	**0.003**	**0.081**	**0.006**

**Abbreviations** DER – Difficulties in Emotion Regulation

*Adjusted effect estimates controlled for age, sexual orientation, race, education, employment, and income

**Bolded** estimates are statistically significant at *p* < 0.05

## Discussion

This study aimed to examine the association between CSA, one of the most well-established correlates of risky sexual behavior in adulthood [49] and CSB, as well as the potential mediating roles of depressive symptoms and difficulties in emotion regulation in this relationship. To our knowledge, this is the first study to examine these mediating pathways among MSM newly diagnosed with HIV.

We found a statistically significant association between CSA and CSB, which aligns with the current literature. This relationship has been demonstrated among varying populations including women, adolescent boys, MSM, and men living with HIV [5, 49–51]. Emetu et al., noted that victims of CSA involving penile–anal intercourse reported sexual behaviors such as a hypersexual self-definition, an STI diagnosis and non-condom use history, and a third sexual partner during sexual activity [52]. Sexual compulsivity has also been linked to overlapping behavioral health and psychosocial problems for MSM [12].

Depressive symptoms as a precipitating factor for CSB has been found in previous studies. Among MSM, research has found links between sexually compulsive behaviors and mood disorders such as depression and anxiety [14, 21], and dissociation [22]. Additionally, research has shown that men with sexually compulsive behavior are more likely to have lower self-esteem [19, 23]. Further studies showed that MSM experience higher levels of depression compared to their heterosexual counterparts [24–28]. Research has also shown that CSA is linked to depression in the general population and also among MSM [32, 33].

The current study found that depressive symptoms and difficulties in emotion regulation fully mediated the association between CSA and CSB. After considering these mediators in the model, the relationship between CSA and CSB attenuated to non-significance. We also found that depressive symptoms correlated with difficulties in emotion regulation. Men who have experienced CSA tend to use maladaptive versus adaptive coping [43].

There are some limitations to be considered in light of the study’s findings. Data were cross-sectional so the temporal sequence between mediators and outcome cannot be established. However, questions regarding CSA referred to events that occurred before the age of 18 and thus a temporal sequence of CSA occurring prior to reported depressive symptoms, emotion regulation, and compulsive sexual behavior can be inferred. Data were self-reported, which may lead to recall bias or social desirability bias. These reports may lead to underestimates or overestimates of the true association. In addition, depression was not diagnosed by a clinician but based on self-reported depressive symptomatology as measured by the CES-D. Future research may assess current, recurrent, and/or lifetime rates of major depressive disorder as mediators. The study population was limited to MSM in New York City and results may not be generalizable to MSM in other geographic locations such as the Southern US or other global MSM populations. However, this study does shed light on the mediating role of depressive symptoms and difficulties in emotion regulation between CSA and CSB among newly HIV diagnosed MSM.

## Conclusion

The study showed that depressive symptoms and difficulties in emotion regulation mediated the association between CSA and CSB among MSM newly diagnosed with HIV. Based on our findings, we believe that a trauma-informed approach to care that recognizes the high prevalence of trauma among those living with HIV and aims to structure the care environment to prevent re-traumatization is central to optimizing care and increasing engagement [53, 54]. Trauma informed care emphasizes the values of (1) ensuring physical and emotional safety; (2) establishing trust and transparency; (3) maximizing individual choice and control; (4) building collaboration and leveling power differences; (5) empowering individuals by recognizing strengths and experience; and (6) acknowledging cultural, historical and gender issues [55]. Screening for sexual violence and CSA in services for MSM may help identify men in need of additional mental and sexual health services. These services may include interventions addressing an individual’s history of CSA, depressive symptoms and difficulties in emotion regulation that may help to reduce CSB among MSM with newly diagnosed HIV who experienced CSA. Further, programs focused on HIV pre-exposure prophylaxis (PrEP), sexual health and sexual risk reduction (e.g., condom use) may be offered to men who have experienced CSA. Future research may include assessing alternative mediators between CSA and CSB, for example, substance use/abuse, and posttraumatic stress disorder, and examining CSA and CSB among older adults living with HIV. Longitudinal research aimed at identifying causal pathways and mechanisms may allow for better tailoring of interventions to reduce mental health problems and risky sexual behavior. Finally, developing or adapting trauma-informed care approaches to address the mental and sexual health needs of survivors of sexual abuse is an important research direction.

## Data Availability

Data can be obtained by e-mailing Dr. Nathan Hansen (nhansen@uga.edu).

## Code Availability

Codes can be obtained by e-mailing Dr. Monique J. Brown (brownm68@mailbox.sc.edu).
